# Concomitant Immunity Induced by Persistent *Leishmania major* Does Not Preclude Secondary Re-Infection: Implications for Genetic Exchange, Diversity and Vaccination

**DOI:** 10.1371/journal.pntd.0004811

**Published:** 2016-06-28

**Authors:** Michael A. Mandell, Stephen M. Beverley

**Affiliations:** Department of Molecular Microbiology, Washington University School of Medicine, St. Louis, Missouri, United States of America; US Food and Drug Administration, UNITED STATES

## Abstract

**Background:**

Many microbes have evolved the ability to co-exist for long periods of time within other species in the absence of overt pathology. Evolutionary biologists have proposed benefits to the microbe from ‘asymptomatic persistent infections’, most commonly invoking increased likelihood of transmission by longer-lived hosts. Typically asymptomatic persistent infections arise from strong containment by the immune system, accompanied by protective immunity; such ‘vaccination’ from overt disease in the presence of a non-sterilizing immune response is termed premunition or concomitant immunity. Here we consider another potential benefit of persistence and concomitant immunity to the parasite: the ‘exclusion’ of competing super-infecting strains, which would favor transmission of the original infecting organism.

**Methodology / Principle Findings:**

To investigate this in the protozoan parasite *Leishmania major*, a superb model for the study of asymptomatic persistence, we used isogenic lines of comparable virulence bearing independent selectable markers. One was then used to infect genetically resistant mice, yielding infections which healed and progressed to asymptomatic persistent infection; these mice were then super-infected with the second marked line. As anticipated, super-infection yielded minimal pathology, showing that protective immunity against disease pathology had been established. The relative abundance of the primary and super-infecting secondary parasites was then assessed by plating on selective media. The data show clearly that super-infecting parasites were able to colonize the immune host effectively, achieving numbers comparable to and sometimes greater than that of the primary parasite.

**Conclusions / Significance:**

We conclude that induction of protective immunity does not guarantee the *Leishmania* parasite exclusive occupation of the infected host. This finding has important consequences to the maintenance and generation of parasite diversity in the natural *Leishmania* infectious cycle alternating between mammalian and sand fly hosts.

## Author Summary

Transmission is an essential aspect in the life cycle of obligate pathogens, and the ability of a pathogen to be transmitted while simultaneously limiting the chances for other pathogens of the same species could provide an important selective advantage. One mechanism whereby a pathogen could accomplish this is by co-opting its host’s immune response to prevent super-infecting pathogens from becoming established, thus effectively gaining exclusive transmission rights from that host. Several pathogen species have evolved the ability to persist indefinitely within their hosts despite the host’s acquisition of protective immunity against subsequent infection by that pathogen, a condition known as concomitant immunity. We asked whether ‘exclusivity’ was a force underlying the evolution of concomitant immunity using the protozoan parasite *Leishmania major* as a model. Using genetically marked parasite lines derived from the same *Leishmania* strain, we show that prior infection does not prevent the entry and survival of subsequently infecting parasites, even though the pathology induced by the secondary infections was markedly reduced. Thus, while persistent *Leishmania* parasites may vaccinate their hosts against pathology, they do not confer protection against superinfection. This finding has important consequences to the maintenance and generation of *Leishmania* genetic diversity, especially through sexual processes.

## Introduction

Persistent host/pathogen relationships are often characterized by a ‘stalemate’ in which the host neither succumbs to disease nor is able to completely achieve sterile cure. Persistent infections can show varying degrees of pathology, ranging from chronic overt disease to asymptomatic infections, reflecting different mechanisms of disease tolerance [1,2,3,4,5,6]. For asymptomatic persistent infections, often a key component is a strong immune response on the part of the host, which is required to keep pathogen numbers in check. In some cases, this immune response also serves to protect against pathology resulting from subsequent re-infection by the same pathogen, a process known as premunition or concomitant immunity [7,8,9].

Long-term host/pathogen relationships carry benefits and risks to both partners, and have been the subject of considerable study from an evolutionary perspective [10,11,12]. In the case of concomitant immunity, the host benefits by its immune system’s ability to control the infection and minimize pathology, as well as protection from disease arising from new infections. However, this comes at the cost of increased risk of disease reactivation, typically following immunosuppression or stress [1,4,13,14,15]. From the pathogen’s perspective, while concomitant immunity decreases microbial numbers, it may improve the likelihood of transmission due to the increased longevity of the infected host.

A second potential benefit to the pathogen of concomitant immunity is ‘exclusivity’, in that the pathogen may use its host’s immune response to gain a competitive advantage by reducing the invasion of the host by other strains or species. For *Schistosoma mansoni*, concomitant immunity may limit intraspecific competition for limited resources [7,16]. This question has been less studied in microbes, where potentially, concomitant immunity could completely or partially preclude secondary colonization of the infected host, and thereby favor transmission of the primary infecting strain. In some respects concomitant immunity might act as a barrier to superinfection in a manner analogous to the mechanisms employed by lysogenic bacteriophages which generally render their bacterial host resistant to super-infection with closely related phage [17]

The protozoan parasite *Leishmania major* provides an excellent model for investigating forces of concomitant immunity and persistence. *L*. *major* is transmitted to mammalian hosts by the bite of phlebotomine sand flies, and in laboratory mice a range of pathology ensues depending on both the particular parasite and mouse strain [18]. Infections of genetically susceptible mice (such as BALB/c) with most *L*. *major* strains yields a progressive and fatal infection [18]. In contrast, infection of genetically resistant mice (such as C57BL/6) initially gives rise to a progressive parasitemia and lesion pathology at the site of inoculation similar to that seen in BALB/c mice, but after 4–6 weeks an immune response develops which controls both parasitemia and pathology [18,19]. Notably, the healed mice are effectively vaccinated and resistant to disease pathology from subsequent infections. Following healing, and for the remainder of the host’s life, a small number of parasites often persists in the skin at the site of inoculation and in the regional lymph node draining that site [20]. In keeping with concomitant immunity/premonition paradigm, these persistent parasites appear to be important for the maintenance of an anti-*Leishmania* immune response, as treatment resulting in sterile cure is associated with the loss of immunity [21,22]. Indeed, the strong protective immunity induced by persistent *Leishmania* is the basis for the ancient practice of leishmanization, in which live, virulent parasites are intentionally inoculated in inconspicuous sites of the body to protect against natural infection and pathology at other sites [23]. However, persistent *Leishmania* are likewise the source for reactivation following immunosuppression [14,15].

Asymptomatic persistent *Leishmania* infections of C57BL/6 mice fit several criteria relevant to understanding of the benefits and tradeoffs of concomitant immunity. The animals are healthy, and despite the small numbers (< 1000 / mouse), persistent parasites can be efficiently transmitted to sand flies [24,25,26]. Several previous studies exploring the immune response induced by persistent parasites inoculated *L*. *major* into a primary site, waited for the lesion pathology to resolve, and inoculated a challenge at a secondary site [22,27,28,29,30]. Each time, viable parasites were recovered from the secondary site, the assumption being that these arose from the secondary challenge. However, *L*. *major* is known to traffic to sites distant from the site of inoculation [20]. Thus, parasites isolated at the secondary inoculation site may have actually originated from the primary infection, perhaps accentuated by the transient reactivation of parasites at the primary infection site as reported by Mendes *et al* [27].

To unambiguously establish the question of secondary colonization and exclusivity, we generated parasites derived from the same strain of *L*. *major* of comparable virulence but bearing independent drug resistance markers (*PHLEO*/phleomycin and *SAT*/nourseothricin). These were then used in the classic infection/challenge persistence model, using one strain as the primary infection, which gave rise to the expected lesion/healing/persistence phenomenon, followed by injection with the second strain in the opposite foot. The results show clearly that under these conditions *Leishmania* persistence is not accompanied by ‘exclusivity’, in that similar numbers of both ‘primary’ and ‘secondary’ parasites persisted at their respective sites of inoculation. These data suggest that while persistent *L*. *major* vaccinates its host from disease pathology, it does not confer exclusivity to the acquisition of secondary infecting *Leishmania*. This finding has important consequences to the maintenance and generation of *Leishmania* genetic diversity, including that arising through sexual processes [31,32].

## Materials and Methods

### Parasite strains and culture

The generation of both the phleomycin resistant parasites (*SSU*:*IR1PHLEO-YFP*; referred to here as LmjF-PHLEO) and the nourseothricin-resistant parasites (SSU:SAT-TK-LUC; referred to here as LmjF-LUC-SAT) used in this study was described previously [33,34]. Parasites were grown at 26˚C in M199 medium (US Biologicals) supplemented with 40 mM 4-(2-hydroxyethyl)-1-piperazine-ethanesulfonic acid (HEPES) pH 7.4, 50 μM adenosine, 1 μg ml^−1^ biotin, 5 μg ml^−1^ hemin, 2 μg ml^−1^ biopterin and 10% (v/v) heat-inactivated fetal calf serum [35]. Nourseothricin (Jena Bioscience, Jena, Germany) was used at a concentration of 100 μg/ml and phleomycin (Sigma, St. Louis, MO) was used at a concentration of 20 μg/ml. Infective metacyclic-stage parasites were recovered using the density gradient centrifugation method [36].

### Ethics statement

This study was carried out in strict accordance with the recommendations in the Guide for the Care and Use of Laboratory Animals of the United States National Institutes of Health. Animal studies were approved by the Animal Studies Committee at Washington University (protocol #20090086) in accordance with the Office of Laboratory Animal Welfare's guidelines and the Association for Assessment and Accreditation of Laboratory Animal Care International.

### Mouse infections

Female C57Bl/6J mice (Jackson Labs) were injected subcutaneously in a hind footpad with 10^5^ metacyclic stage parasites. Naïve mice (6–8 weeks old) were injected in the left hind footpad. Secondary injections took place in the right hind footpad at a time point >1 month after primary lesions had resolved. Footpad lesion thickness was measured using a Vernier caliper (Mitutoyo). Lesion size was calculated as the difference in thickness between the infected and uninfected footpads. Luciferase activity was determined as described elsewhere [34]. Briefly, mice were given a dose of D-luciferin (150 μg gram^-1^ body weight; Biosynth) in PBS 10 minutes prior to imaging with an IVIS 100 imaging system (Xenogen Corp). In this study, values less than 105 p/s fall into the background range. Limiting dilution assays were performed as described previously [37], with the addition of phleomycin or nourseothricin as indicated. Reconstruction experiments suggest that the limit of detection was about 14 parasites/footpad.

### Statistics

Data are presented as the arithmetic mean ± the standard deviation. *P* values were calculated by the Student’s t-test.

## Results

### Development of two genetically marked *L*. *major* with comparable virulence in resistant mice

**Fig 1:**
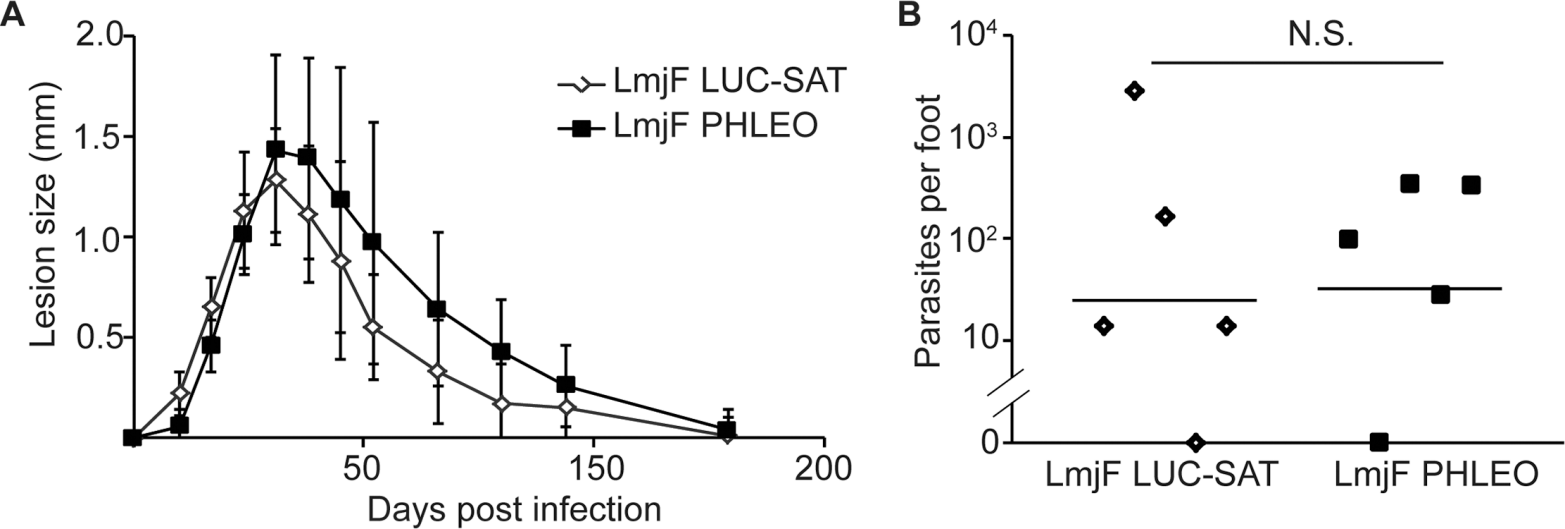
The LmjF-LUC-SAT and LmjF-PHLEO strains show comparable virulence in infections of resistant mice. C57BL/6 mice (5 per group) were infected with 10^5^ metacyclic stage LmjF-LUC-SAT or LmjF-PHLEO *L*. *major*. (A) Measurements of lesion pathology (increase in footpad thickness). Error bars show the standard deviation. (B) Persistent parasites numbers were determined by limiting dilution assay from footpad tissue 130 days post infection. Horizontal bars show the geometric mean. †, *P* > 0.05.

We used two *L*. *major* Friedlin V1 parasites expressing genes conferring resistance to the antibiotics nourseothricin (*SAT*) or phleomycin (*PHLEO*). The nourseothricin resistant parasites also express firefly luciferase, and will be referred to hereafter as “LmjF-LUC-SAT”, while the phleomycin resistant parasites will be referred to as LmjF-PHLEO. To confirm that the LmjF-LUC-SAT and LmjF-PHLEO parasites were of comparable virulence in mice, 10^5^ metacyclic-stage parasites were inoculated into the footpads of naïve C57BL/6 mice (5 mice/group), and the lesion pathology was monitored over time (Fig 1A). Both lines exhibited disease progression typical of untransfected *L*. *major* / C57BL/6 infections, with lesions developing between 10–17 days post infection and reaching their maximum (~1.4 mm increased footpad thickness) around 30 days post infection [38,39]. Thereafter the lesions declined, and were completely resolved by 130 days post-infection (Fig 1A). While there was some tendency for the LmjF-LUC-SAT line to show smaller lesion sizes, at no point was this difference statistically significant. After resolution, mice were sacrificed and the parasite titers in the infected feet were enumerated by limiting dilution analysis (Fig 1B). The number of persistent parasites recovered for both lines was in agreement with what is expected in this experimental system (typically 100–1000 parasites, with substantial variability amongst mice and experiments) [20,27,28,30,40]. Importantly, we found no significant difference in the number of persistent parasites between the two lines, with LmjF-LUC-SAT and Lmj-PHLEO showing a similar range (Fig 1B) and mean (25 and 32 parasites / foot; *P* > 0.45 by Student’s t-test). We judged these lines to be of comparable virulence and suitable for subsequent experiments.

### Healed mice were protected against pathology from subsequent challenge

Two experiments were performed in which naïve mice (4–5 mice per experiment) were inoculated with 10^5^ purified metacyclic-stage LmjF-LUC-SAT parasites in the left hind footpad primary infection site. A lesion formed at that site and resolved in accordance with the data shown in Fig 1A. At a time point >1 month after resolution (1.5 and 3 months for experiments 1 and 2 respectively), 10^5^ metacyclic LmjF-PHLEO parasites were inoculated into the right hind footpad secondary infection site. Footpad swelling of both the primary (L) and secondary (R) injection sites was then measured over time. We also used *in vivo* imaging of luciferase activity to visualize LmjF-LUC-SAT parasites, as a second probe of whether transient reactivation of primary parasites occurred [27].

**Fig 2:**
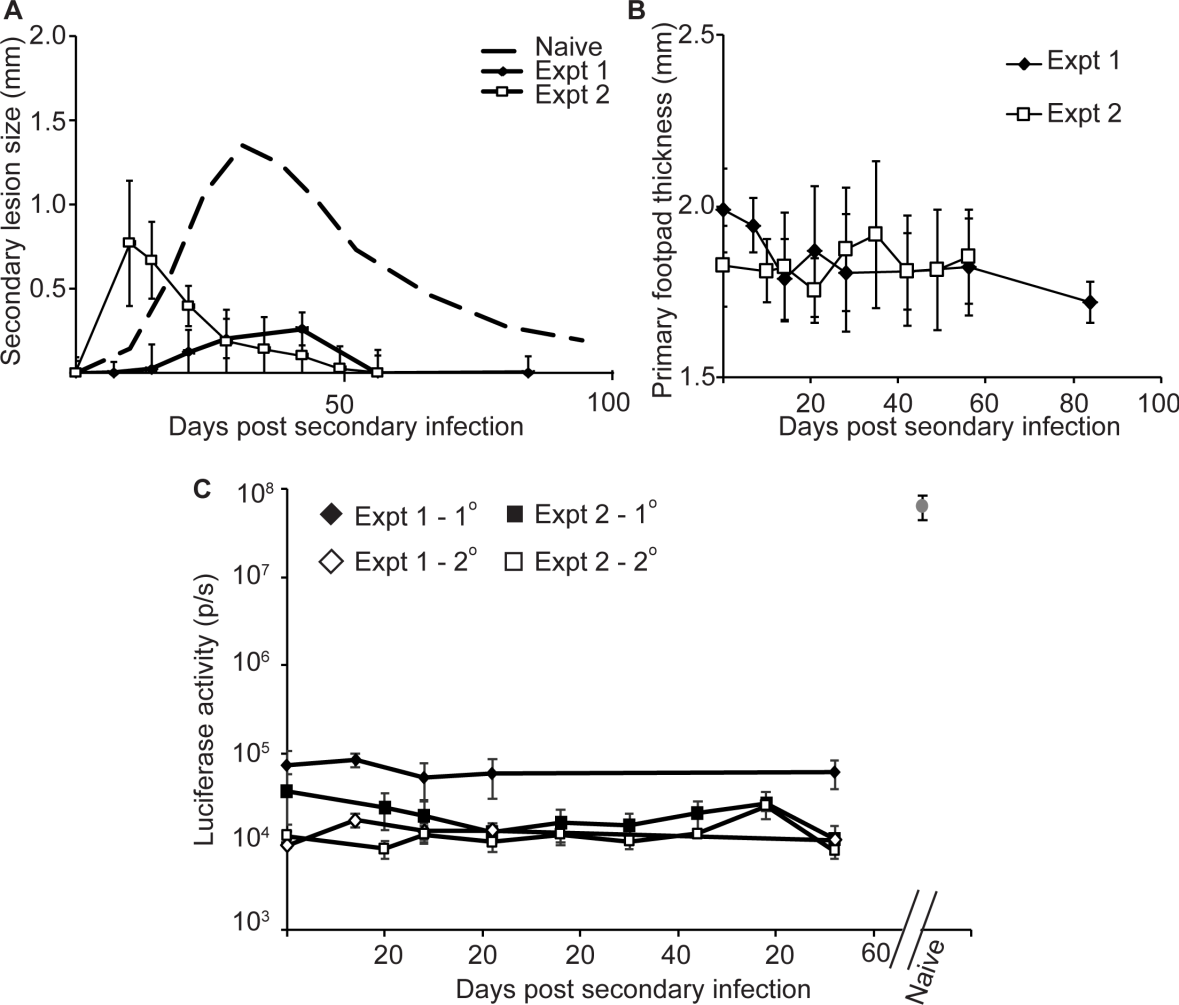
Mice persistently infected with LmjF-LUC-SAT show protection from disease pathology by secondary challenge with LmjF-PHLEO parasites. Mice (4-5/group) were inoculated with 10^5^ metacyclic LmjF-LUC-SAT parasites in the left hind footpad (primary site), after which they developed lesions and then went on to heal (similar to that shown in Fig 1A). (A) At least one month after resolution of the primary lesions, each mouse was inoculated in the right hind footpad (secondary site) with 10^5^ metacyclic LmjF-PHLEO parasites, and lesion progression is shown in the figure. The dashed line represents the average of the data presented in Fig 1A for infections of naïve mice with LmjF LUC-SAT and LmjF-PHLEO for comparison. In these experiments “time 0” is when the secondary inoculation was performed unless otherwise indicated. For all plots, error bars show the standard deviation (n = 4 or 5 in expt. 1 or 2 respectively). (B) Footpad thickness at the primary injection site (left foot). (C) Monitoring of reactivation of the primary LmjF-LUC-SAT parasites at the primary (♦,■) or secondary (◊,□) infection sites site by bioluminescent imaging of luciferase expression *in vivo*; experiment 1 (♦,◊); experiment 2 (■,□). The gray circle (upper right) shows the luminescence profile of LmjF-LUC-SAT parasites infecting naïve mice at the peak of infection, included for comparison only. Error bars depict the standard deviation.

As expected, in both experiments the mice showed good protection, as evidenced by a reduction in lesion pathology at the secondary ‘challenge’ site. Although with some variation, in both experiments the lesions generated by the secondary LmjF-PHLEO parasites were significantly smaller and resolved more rapidly than those in naïve mice (Fig 2A). We saw no evidence of reactivation of the “primary” LmjF-LUC-SAT parasite, as judged by either lesion measurement (Fig 2B) or *in vivo* imaging of parasite luciferase (Fig 2C, left), the latter yielding values in the background range, and orders of magnitude less than what is seen following infection of naïve mice by these parasites at the peak of parasitemia (Fig 2C, right).

### Similar numbers of both “primary” and “secondary” parasites persist

**Fig 3:**
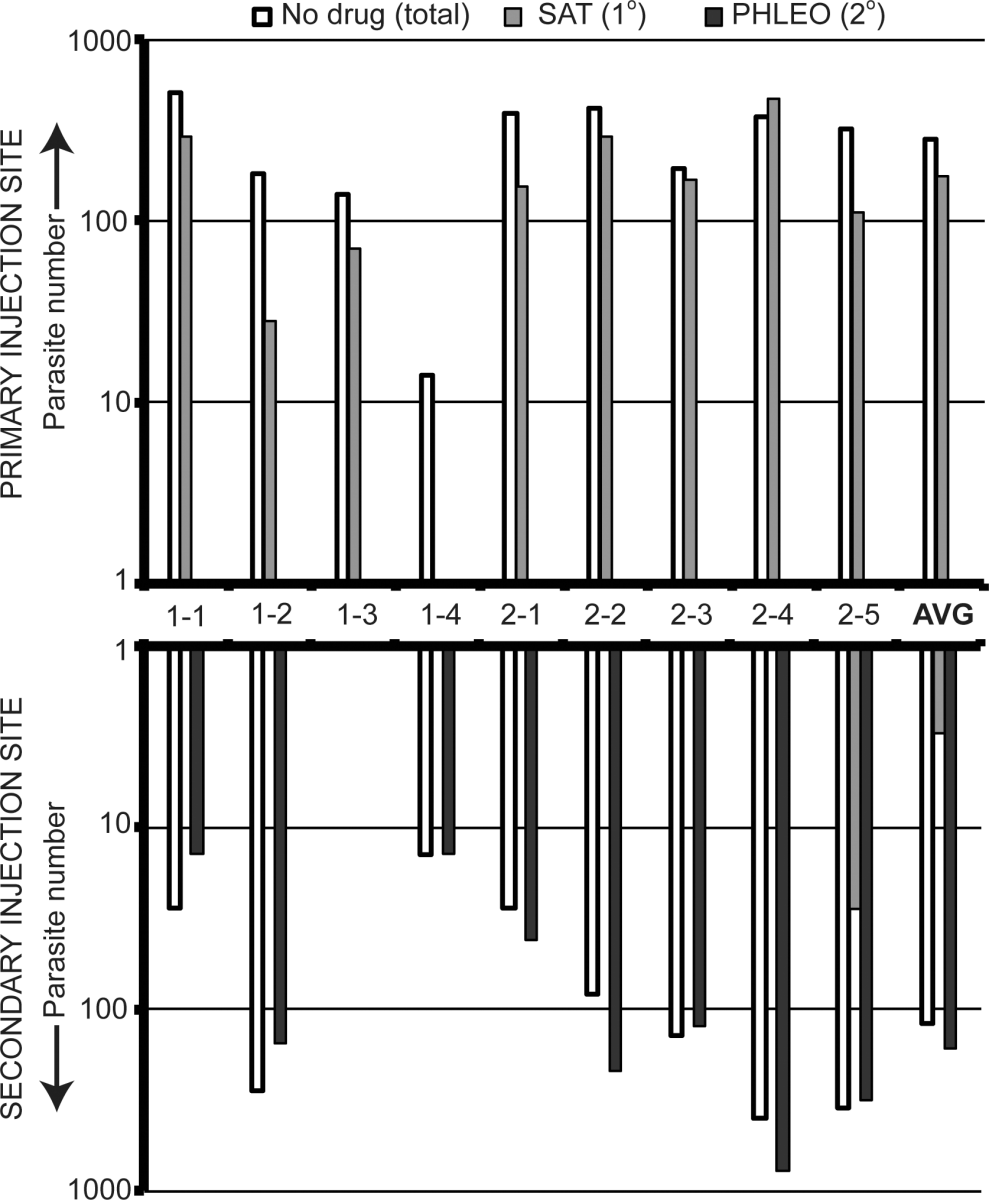
Retention of both primary and secondary infecting parasites following secondary challenge despite protection from disease pathology. The graph plots the number of persistent parasites present in sites of primary and secondary *Leishmania* infections >10 weeks post secondary challenge as assessed by limiting dilution analysis in unselective (white bar), nourseothricin-containing (gray bars; resistance mediated by *SAT* marker) or phleomycin-containing (black bars; resistance mediated by *PHLEO* marker) as described in the methods. The number of parasites in the primary infection site (LmjF-LUC-SAT inocula) is displayed in the top graph, and the number of parasites in secondary infection site (LmjF-PHLEO inocula) foot is displayed in the bottom graph. The numbers between the two graphs represent the mouse identification number (experiment number-mouse number). “Avg.” represents the mean for all mice.

Having established the classic *Leishmania* paradigm of vaccination following resolution of a primary challenge for the genetically marked lines in our study, we then measured the occurrence of both the primary- and secondary- infecting parasites, in both infection sites. This was performed by limiting dilution assays at day 87 (experiment 1) or day 139 (experiment 2) post-infection. Total parasites were assessed by growth in the absence of drug, while LmjF-LUC-SAT (primary) was estimated from growth in media containing nourseothricin and LmjF-PHLEO (secondary) from growth in media containing phleomycin. The results from individual mice from both experiments as well as the global averages are shown in Fig 3.

Parasites were recovered from all primary infection sites, ranging from 14 to 504 parasites/foot, with an average of 282 ± 158 parasites recovered per foot (N = 9). These parasites were exclusively the primary LmjF-LUC-SAT parasite, as they were unable to grow in the presence of phleomycin. In one animal parasites expressing the SAT marker were apparently lost; similar results have been reported in *L*. *tarentolae* and attributed to the genetic plasticity of the ribosomal RNA locus [41], and we have seen this occasionally in other experiments in *L*. *major*.

Parasites were also recovered from the secondary infection site from 8 of the 9 mice, ranging from 14 to 785 parasites/foot, with an average of 119 ± 156 parasites/foot. Importantly, nearly all of the parasites recovered from the secondary infection site were the LmjF-PHLEO parasite inoculated there (99 ± 3%). In only one mouse (#2–5) was colonization of the secondary site by ‘primary’ infection site LmjF-LUC-SAT parasites found, suggesting that metastasis of parasites from the primary to the secondary sites occurs infrequently. Importantly, the numbers of ‘primary’ infection site LmjF-LUC-SAT parasites were not significantly different from that seen for the ‘secondary’ infection site LMjF-PHLEO parasites (*P* > 0.08, Student’s T-test). These data show that despite successful ‘vaccination’, as defined by reduction in lesion pathology, this immunity was not ‘sterilizing’ against secondary infection and did not preclude efficient colonization of the infected mouse significantly.

## Discussion

A number of factors have been proposed to contribute to the maintenance of pathogens for long periods of time in the host, including an insufficient immune response and the benefits accruing to the pathogen from residing within a longer-lived host thereby increasing the likelihood of transmission [10,12]. In many cases this relationship has progressed to the point where the pathogen infection is asymptomatic, thereby fulfilling the evolutionary dictum that a ‘successful pathogen does not kill its host too quickly”. Often this asymptomatic persistence is accompanied by protection from disease induced by further infections of the same or related pathogens, a process termed concomitant immunity [7]. Such a relationship provides benefits to both the pathogen and the host through increased longevity of the latter (albeit with some risk of reactivation), and increased transmission of the former.

*Leishmania* provides an attractive system for the study of concomitant immunity [20,21,27,42,43,44] and here we have used this to consider another potential benefit to the pathogen, one of ‘exclusivity’. Exclusivity would favor transmission of the primary infecting pathogen due to reduction in the ability of secondary infecting parasites to becoming established in a previously infected host. However, our data show clearly that despite induction of a protective immune response able to mitigate disease pathology (Fig 2), secondary *Leishmania major* infections are nonetheless able to establish themselves effectively in a previously infected host (Fig 3). While this result may have been anticipated from prior studies [22,28], this is the first time this has been established rigorously for *Leishmania* using genetically marked parasites able to distinguish primary from secondary infections and bioluminescent imaging to assess reactivation. Our studies also provide limited support for the prior assumption that in general parasites are not frequently transferred from the primary to the secondary site of infection, although we did observe transfer in one mouse (Fig 3, mouse #2–5).

Consistent with prior studies, the immunity generated by persistent parasites was not always sterilizing and the average number of “secondary” parasites was not statistically different from that of the “primary” parasites (Fig 3). Nonetheless, the average number of parasites recovered from the secondary site was about 2-fold less than from the primary site, similar to the findings of Mendez *et al* (2004) [27]. Thus, it is possible that secondary infecting parasites may experience a modest quantitative disadvantage, which over evolutionary time could provide a strong positive selective force on the parasite favoring the induction of concomitant immunity. This phenomenon may warrant further study in the future.

In our studies an inoculum of 10^5^ purified metacyclic parasites was used. While most sand flies transmit less than 600 parasites to mice, some transmit up to 10^5^ [25]. Thus, the infecting dose used here falls on the high side of the biologically relevant range. Studies using low-dose infections with 100 metacyclics also recovered parasites from the site of secondary infection, although genetic markers were not available to confirm their identity [22,27,28,29]. Undoubtedly there are a number of experimental variables that could be pursued in future studies, including infecting dose, the relative timing of the primary and challenging infections, or sites of inoculation other than the footpad that may be potentially relevant, such as the ear, snout or tail. Another important variable is the extent of genetic identity between the primary and secondary infections; we purposefully chose to study isogenic parasite lines here to maximize the likely efficacy of concomitant immunity, the efficacy of which might be expected to decrease with heterologous strains or event species. Lastly, while our experiments were carried out in an ‘orderly’ manner with primary and secondary infections in separate feet, nature is decidedly less so, and indeed infections may occur at the same location [45], thereby increasing the likelihood of transmissible mixed infections.

An important question is the relevance of ‘needle’ infections performed here to natural sand fly transmission, where parasites are deposited along with immunomodulatory factors of both sand fly and parasite origin. These factors include saliva and secreted parasite molecules such as proteophosphoglycan, both of which typically act to facilitate primary infections [46,47,48,49] but which can also engender various protective responses [50,51] and thus have the potential to either favor or hinder the entry of the secondary ‘invading’ *Leishmania*. In several studies examining challenge by sand fly bite of mice which had healed from primary infections, sterilizing immunity was seen in 33/64 mice tested in challenge infections (52%), while the remainder showed minimal pathology accompanied by parasite numbers ranging from 100 to 10,000 at the challenge bite site [48,52]. Assuming that these parasites arise primarily from the challenge parasite (as shown here), both natural sand fly and ‘needle’ challenge can yield infections with robust parasite survival at the secondary challenge site at significant frequencies.

### Consequences of ‘nonexclusive’ parasitism to parasite diversity and vaccination strategies

That concomitant immunity induced by primary *L*. *major* infections protects against pathology can occur at significant frequencies without sterilization, instead leading to ‘mixed’ infections of the host, has important implications for the generation and maintenance of *Leishmania* diversity. In regions where *Leishmania* is endemic, mammalian hosts are likely subjected to many bites by infected sand flies [53,54], which over time could result in the host being persistently infected with several genetically distinct parasite lines. There are numerous reports documenting the recovery from infected animals and humans of *Leishmania* stabilates exhibiting mixed genotypes, using a variety of molecular taxonomic methods [55]. Some fraction of these represent true mixed infections, while others may arise from the presence of intra- or inter-specific hybrids [31,55,56,57,58,59,60]. In several studies the incidence of mixed populations exceeded 10% [54,61,62,63]. Moreover, concerns have been raised about the efficiency of detection of mixed infections, ranging from technical analysis to problems associated with differential outgrowth during adaptation to culture [62,63,64], suggesting that the true incidence may be greater than presently appreciated. Notably, human infections showing overt pathology have been most highly sampled, and even for humans the situation in the more prevalent ‘asymptomic’ infections (primary or secondary) is largely unknown. Thus while it is difficult to say with any certainty what fraction of natural *Leishmania* infections are truly ‘mixed’ in human or animal reservoir populations, they are far from rare, and potentially quite common.

Once established, mixed infections have the potential to be passed on to sand flies, which have recently been shown to be the site of both intra-specific and interspecific genetic exchange [32,65,66,67,68]. Since the frequency of sand flies bearing *Leishmania* in natural populations is relatively low (often just a few percent) [69,70,71], the accumulation and maintenance of mixed populations over time in persistent mammalian infections would act to increase the frequency at which sand flies acquire mixed infections, which thereafter undergo genetic exchange and generate diversity. While genetic exchange occurs relatively infrequently on a per *Leishmania* cell basis (<10^−4^; [32]), *Leishmania* numbers in sand flies are sufficient to yield hybrid parasites at high frequencies (25% or greater per fly; [32,65]. Thus, the lack of ‘exclusivity’ even in the presence of protection against disease pathology may result in increased opportunities for genetic exchange and the emergence of new disease phenotypes in nature [72].

Our data also have some consequences to vaccination strategies. Currently the ‘healed’ mouse is considered a ‘gold standard’ for the maintenance of effective immunity against disease pathology, and the generation of live-attenuated parasite lines that persist without pathology while immunizing against virulent challenge has been a priority in vaccine research [40,73]. Our data suggest that such an approach would likely allow virulent parasites from subsequent natural infections to establish their own persistent infections, which could then pose a risk of reactivation and/or transmission. This may provide further impetus for the development of vaccines conferring sterilizing, long-lasting protection against both pathology and parasitemia.
